# Health benefits of electrically-assisted cycling: a systematic review

**DOI:** 10.1186/s12966-018-0751-8

**Published:** 2018-11-21

**Authors:** Jessica E. Bourne, Sarah Sauchelli, Rachel Perry, Angie Page, Sam Leary, Clare England, Ashley R. Cooper

**Affiliations:** 1 Centre for Exercise, Nutrition and Health Sciences, School of Policy Studies, University of Bristol, 8 Priory Road, Bristol, BS8 1TZ UK; 2 NIHR Bristol Biomedical Research Centre, University Hospitals Bristol NHS Foundation Trust and University of Bristol, Bristol, UK

**Keywords:** Electrically-assisted bicycle, E-bike, Physical activity, Health

## Abstract

**Background:**

Electrically assisted bicycles (e-bikes) have been highlighted as a method of active travel that could overcome some of the commonly reported barriers to cycle commuting. The objective of this systematic review was to assess the health benefits associated with e-cycling.

**Method:**

A systematic literature review of studies examining physical activity, cardiorespiratory, metabolic and psychological outcomes associated with e-cycling. Where possible these outcomes were compared to those from conventional cycling and walking. Seven electronic databases, clinical trial registers, grey literature and reference lists were searched up to November 2017. Hand searching occurred until June 2018. Experimental or observational studies examining the impact of e-cycling on physical activity and/or health outcomes of interest were included. E-bikes used must have pedals and require pedalling for electric assistance to be provided.

**Results:**

Seventeen studies (11 acute experiments, 6 longitudinal interventions) were identified involving a total of 300 participants. There was moderate evidence that e-cycling provided physical activity of at least moderate intensity, which was lower than the intensity elicited during conventional cycling, but higher than that during walking. There was also moderate evidence that e-cycling can improve cardiorespiratory fitness in physically inactive individuals. Evidence of the impact of e-cycling on metabolic and psychological health outcomes was inconclusive. Longitudinal evidence was compromised by weak study design and quality.

**Conclusion:**

E-cycling can contribute to meeting physical activity recommendations and increasing physical fitness. As such, e-bikes offer a potential alternative to conventional cycling. Future research should examine the long-term health impacts of e-cycling using rigorous research designs.

**Electronic supplementary material:**

The online version of this article (10.1186/s12966-018-0751-8) contains supplementary material, which is available to authorized users.

## Background

Given the high rates of global physical inactivity [1] a growing body of research has focused on the potential of active travel to increase physical activity behaviour and potentially lead to population health benefits. Engagement in active travel, specifically commuting, has been shown to be predictive of a lower BMI [2] and reduced risk of diabetes diagnosis [3]. A recent prospective study reported that active commuting, involving cycling, was associated with a lower risk of all-cause mortality and cancer incidence and mortality [4]. In addition, commuting by bicycle or on foot was associated with a lower risk of cardiovascular disease incidence and mortality [4]. The greatest gains in health outcomes from active commuting are reported in the least active individuals [5, 6].

Travel is an essential part of everyday life for most people, and the adoption of active travel represents an efficient way to increase daily physical activity. For example, Falconer and colleagues [2] found that active commuting was associated with an additional 73 weekly minutes of moderate to vigorous physical activity in men and 105 weekly minutes in women with type 2 diabetes, compared to those commuting using motorised transport. With half of all car journeys in the UK being between 1 and 5 miles in length [7], the substitution of many car journeys by walking and/or cycling may be an achievable aim.

Due to a growing body of evidence, the UK National Institute of Health and Care Excellence (NICE) now endorse active travel, with a particular focus on commuting, as a feasible method to incorporate physical activity into daily life [8]. However, rates of active commuting are low [9].Common barriers to cycle commuting include the physical constraints associated with hilly terrain, poor physical fitness, lack of time and the distance to work [10].

Electrically assisted bicycles (e-bikes) have been highlighted as an alternative method of active travel that could overcome some of the commonly reported barriers to cycle commuting [11]. The term e-bike includes a range of designs including throttle-controlled bikes which do not require the rider to pedal and electrically assisted bikes which provide electrical assistance only when the rider is pedalling, through sensors which detect pedalling speed and force [11]. It is through pedalling that electrically-assisted cycling may serve to increase physical activity. With lower motor power and maximum speeds compared to throttle-controlled e-bikes, electrically-assisted bikes are legally classified as bicycles. [11]. For this review the term e-bike will be used exclusively to refer to electrically-assisted bicycles which require the rider to pedal.

In recent years e-bikes have become commonplace in European countries [11] with projected global sales of 47.6 million by the end of 2018 [12]. E-bikes are increasingly used for both leisure and commuting purposes [13]. The assistance provided has been reported to motivate novice cyclists and increase the likelihood that these individuals will continue to cycle in the future [10]. Given the increasing interest in e-bikes, and their use for active travel, there is a need to understand their potential to promote physical activity of a sufficient intensity to gain clinical benefit (i.e., moderate-to-vigorous intensity [14]) and to examine their impact on broader health outcomes. Such research is required to inform relevant health economic assessments and public health policy. To date, there has been no systematic review on the physical activity intensity and health outcomes associated with e-cycling. As such the aims of this systematic review are to answer the following research questions:

1. What is the intensity of physical activity associated with riding an e-bike?
2. Does use of an e-bike lead to changes in health outcomes including cardiorespiratory, metabolic or psychological outcomes?
3. Do physiological responses to riding an e-bike differ to those generated by other modes of active transportation (i.e. walking and conventional cycling)?

## Methods

A review protocol was registered at the PROSPERO database: Registration number CRD42018086544 (http://www.crd.york.ac.uk/prospero). This review was conducted according to the guidelines outlined by the Preferred Reporting Items for Systematic Reviews and Meta-Analyses guidelines [15].

### Search strategy

The following databases were searched from their inception to November 2017: PsychINFO, MEDLINE and Embase (via Ovid), ISI Web of Science, CINAHL complete, SPORTDiscus and Scopus. Search terms were *‘pedelec’, ‘e-bike’, ‘electrically assisted bicycle’, ‘electrically assisted cycle’, ‘electrically assisted bike’, ‘pedal-assist’, ‘electric bicycle’, ‘electric bike’, ‘electric cycle’, ‘electric mobility’* (see Additional file 1 for example). Reference lists from all selected articles were hand-searched for relevant studies. OpenGrey and Google Scholar (first 20-pages) were searched using the term ‘*electrically-assisted bicycle’.* Hand-searching occurred until June 2018.

### Inclusion criteria and selection process

Studies were eligible for inclusion if they met the following criteria:

1. participants: adults ≥18 years of age,
2. electrically-assisted bicycle must have pedals and be operated by the individual, with assistance available from an electric motor
3. at least one of the following outcomes; objective measure of physical activity intensity whilst e-cycling (e.g., metabolic equivalents, energy expenditure), cardiorespiratory, metabolic or quality of life (as a measure of psychological health),
4. type of study: experimental or observational studies.

Studies could be published or unpublished in any language. For articles in a language other than English the title and abstract were translated using Google Translate. If full text screening was required, the article was translated by an individual fluent in the language. Studies were excluded if they reported using bicycles that did not require the individual to pedal to provide power, were review articles or commentary pieces, and/or used self-reported measures of physical activity. Title and abstract screening was conducted by two reviewers independently (J.E.B. and S.S.). There was a 93% agreement between reviewers on title and abstract screening. Full texts were screened by the two reviewers independently and any discrepancies were discussed.

### Quality assessment and strength of the evidence

The quality of included studies was assessed using the Quality Assessment Tool for Quantitative Studies (EPHPP; [16]). The tool appraises studies on six components; 1) selection bias, 2) study design, 3) control of confounders, 4) blinding, 5) reliability and validity of data collection methods and 6) withdrawals and dropouts. Each component was rated as; strong, moderate or weak for each study based on outcomes of interest.

A global rating for each study was then determined based on the criteria; 1) strong when no weak ratings were reported, 2) moderate when one weak rating was reported, and 3) weak when two or more components were rated as weak. This tool has been used in a previous review examining the impact of cycling on health [6]. The blinding component was not included in the overall study rating as participants are unable to be blinded to condition allocation following randomisation in physical activity interventions. The overall strength of the evidence was assessed based on previously specified best evidence synthesis criteria [17] (Additional file 2).

### Data extraction and synthesis

Members of the review team (J.E.B and either S.S. or A.R.C) independently extracted data for each study. Quality assessment was confirmed by a fourth reviewer (R.P.). Data were extracted using an adapted version of a Cochrane Data Extraction Form, which was piloted prior to use. Discrepancies regarding data extraction were resolved through discussion between reviewers. Data extracted included study design, characteristics of participants, outcomes measured, and results. Due to the heterogeneity of study design and outcomes reported, a meta-analysis was not deemed appropriate. Data were synthesized and presented narratively. The effect of the intervention on physical activity and health outcomes for each study was summarized based on reported statistical significance and effect size, both within group (pre-post) and between group where possible, or by examining means or medians when no hypothesis testing was conducted.

## Results

**Fig. 1:**
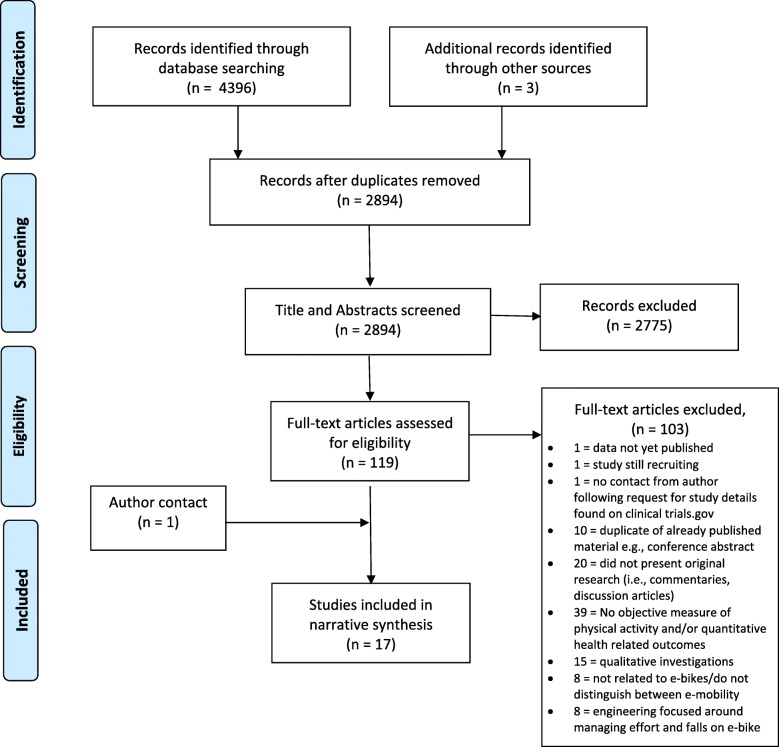
Flow chart of literature search

A total of 4399 articles were identified through initial searches (Fig. 1). After removing duplicates 2894 titles and abstracts were screened, resulting in 119 studies which underwent full text screening for inclusion. Sixteen articles met the criteria for inclusion plus one included after author contact. Eleven studies assessed the acute response to e-cycling (i.e., one bout of e-cycling), and six examined the longitudinal effect of e-cycling (i.e., more than one bout of e-cycling, including pre-post measurements). Reasons for exclusion included no measure of specified outcomes, study not related to e-bikes, studies focused on the engineering of e-bikes, qualitative studies or not presenting original research. Three studies were identified through clinicaltrials.gov but were excluded for the following reasons: 1) data not published, 2) currently recruiting, 3) authors were not reachable.

### Study characteristics

#### Acute studies

Eleven studies examined the acute physiological impact of e-cycling using cross over designs, five of which were randomized (Table 1). Nine studies were conducted in Europe and two in the USA. Sample sizes ranged from 3 to 22 with a total of 147 participants. Participants were aged between 20 and 70. Three studies recruited physically inactive individuals [18–20] and one study included individuals with coronary artery disease [21]. Six studies compared e-cycling to conventional cycling [18, 21–25] and five compared e-cycling with assist to riding an e-bike without assistance [19, 20, 26–28]. Two studies included walking as a comparator [18, 23].

**Table 1:** Summary of included studies

First author, year, country	Study design	Participants; gender (%female); Age, years (mean, SD); BMI, kg/m^2^ (mean, SD)	Clinical status	Exposure conditions	Length of intervention	Ride characteristics Distance (km), Topography, Distinctive features, Ride instructions
Acute studies						
Bernsten, 2017, Norway [22]	Randomized cross over	*N* = 8, 25% Age (*Mdn, IQR):* 39(3) BMI (*Mdn, IQR):* 24(7)	Active adults	E-bike vs. CB (*4 conditions, hilly* vs. *flat terrain)*	Trials conducted on same day, 2-min break between trials	Route 1: 8.1 km, flat route Route 2: 7.1 km, one hill climbed twice 130 m elevation gain. Self-selected intensity
Gojanovic, 2011, Switzerland [18]	Non-randomized cross over	*N* = 18, 33.33% Age: 35.7 (±9.7) BMI: 24.0 (±3.3)	Inactive adults	E-bike LA vs. E-bike HA vs. CB vs. walking	Trials conducted over 2-days. 30-min break between trials conducted on same day	Biking: 5.1 km, 178 m elevation gain, average gradient 3.4% Instructed to ride at comfortable pace maintaining 60 rpm Walking: 1.7 km, uphill, 110 m elevation gain, average grade 6.5%
Hansen, 2017, Belgium [21]	Randomized cross over	*N* = 17, 13% Age: 64 (±7)	Coronary artery disease	E-bikes LA vs. E-bike HA vs. CB	Trials conducted on separate days (3–4 days between)	10 km, 102 m elevation change No traffic or stop and go points Instructed to cycle at self-selected pace on prespecified mode
La Salle, 2017, USA [26]	Randomized cross over	*N* = 12, 50% Age: M = 25(±1), F = 22(±1) Body Fat %: M = 16.8(±1.9), F = 23.4 (±3.3)	Active adults with cycling experience	E-bike pedal assist vs. E-bike NA	Trials conducted in same day. Average time between trials 12-min	3.54 km, hill 0.64 km 11% gradient Seven pedestrian crossings participants required to dismount and walk. Self-selected pace
Langford, 2017, USA [23]	Non-randomized cross over	N = 17, 35% Age: < 20 yrs. = 3, 20-30 yrs. = 10, 31-40 yrs. = 2, > 50 yrs. = 2 BMI: M = 26.1, F = 23.1	Adults, part of e-bike sharing system	E-bike vs. CB vs. Walking	Trials conducted on separate days (minimum 24h rest)	4.4 km, 1.6 km downhill (− 33.2 m), 1.8 km flat (− 0.3 m), 1.0 km uphill (+ 33.5 m). Self-selected pace
Louis, 2012, France [27]	Randomized cross-over	*N* = 20 (10 T, 10 UT) Age: *T* = 38.7 (±14.8); *UT* 28.9 (±6.3) BMI: *T* = 22 (±1.1), *UT* = 22.2 (±3.7)	Highly active adults (T) Recreationally active adults (UT)	E-bike NA vs. E-bike LA vs. E-bike HA	Trials conducted on same day. 5-min breaks between trials	Completed on indoor trainer. Instructed to pedal at specified mode for total of 45-min at pre-specified speeds: 15-min at 16 km/hr., 21 km/hr. and free speed totaling 45-min.
Meyer, 2014, Germany [28]	Non-randomized cross over	*N* = 3, 0% Age: 25, 25, 27 Weight (*Kg):* 74, 71, 79	Active adults, recreational cyclists	E-bike pedal assist vs. E-bike no assist	Trials conducted on separate days, 1-day apart.	27 km track divided in 5 sections
Simons, 2009, Netherlands [20]	Non-randomized cross over	*N* = 12, 50% Age: 52.2 (8.7), range 32–60 BMI: 24.5 (2.6)	42% inactive adults 58% recreationally active adults	E-bike NA vs. E-bike LA vs. E-bike HA	Trials conducted in same day. One-hour rest between trials.	4.3 km, flat route, two stop and go section participants required to dismount and restart. Self-selected pace on pre-specified intensity
Sperlich, 2012, Germany [19]	Randomized cross over	*N* = 8, 100% Age: 38(±15) BMI: 25.3 (±2.1)	Inactive adults	E-bike pedal assist vs. E-bike no assist	Trials conducted in same day. One-hour rest between trials.	1.9 km × 5 = 9.5 km, 200 m uphill 1, 5.9%, 700 m downhill, 300 m uphill 2, 5.8%, 700 m flat. Self-selected pace and gear
Theurel, 2011, France [24]	Non-randomized cross over	*N* = 22, 18% female Age: M = 41(±11), F = 34(±9) Weight (*Kg):* M = 68(±18), F = 76(±10)	Active postal workers	E-bike vs. CB	Trials conducted on same weekday, 1-month apart	Postal route, one group completed rides in residential neighbourhood, the other completed the ride in downtown location
Theurel, 2012, France [25]	Non-randomized cross over	*N* = 10, 50% female Age: F = 30 (±12), M = 35 (±14)	Active adults	E-bike vs. CB	Trials separated by 1 week	30-min of intermittent cycling on inside track alternating cycling of 10 sec duration and recovery of 20 sec. Aimed to complete 60 m in 10 sec (average speed = 21.6 km/hr)
Longitudinal studies						
Cooper, 2018, UK [32]	Single group feasibility	*N* = 20 (report on 18) Age: 58.1 (±7.9) BMI: 30.2 (4.4)	Type 2 Diabetes	One group e-bike	Up to 5 months	E-bike training provided. Provision of e-bike for up to 5-months. Support for mechanical issues provided. No instruction on how or when to ride bike
De Geus, 2013, Belgium [29]	Non-randomized cross over	*N* = 24, 46% Age: M = 47(±7) F = 43(±6) BMI: M = 27.0 (±2.8), F = 24.7 (±4.6)	Inactive adults ^a^	E-bike vs. Control	Control = 4 weeks E-bike = 6 weeks	Instructed to ride e-bike at least three times per week to commute to and from work
Hochsmann, 2017, Switzerland [30]	Pilot randomized controlled trial	*N* = 32, 13% Age, (*Mdn, IQR):* F = 35(34–45), M = 43(38–45) BMI, (*Mdn, IQR)* E-bike = 29 (27,31), regular bike = 28 (26,29)	Inactive adults	E-bike vs. CB	4 weeks	Instructed to use bike for active commute to work on at least 3-days per week, over 6 km. Self-selected pace
Malnes, 2016, Norway [31]	Single group pilot	*N* = 25, 72% Age: 42(±12) BMI: M = 25.4(±12.3), F = 28.7(±15.8)	Inactive adults	One group e-bike	Up to 8 months	3 sites: 2 provided e-bikes for up to 8-months, 1 e-bike up to 3-months. Instructed to use bike as desired. In 2-centres if e-bikes not used they were withdrawn from participant. Group was separated into high and low fitness groups based on baseline testing
Page, 2017, UK [33]	Non-randomized two group	*N* = 31, 80% Age Range: 21-55 years	Unclear	E-bike commuting vs. passive commuting	Data reported mid-way into intervention – 2 months	No instructions on how to ride bike, full roadside assistance provided.
Peterman, 2016, USA [13]	Single group	*N* = 21, 70% (of 20 in analysis) Age: 41.5 (±11.5).	Inactive adults	One group e-bike	4 weeks	Instructed to ride e-bike at least 3 days per week for at least 40-min for commuting

*T* trained (engage in endurance sport at least 4 times per week), *UT* untrained (moderately active but less than 4× per week), *Inactive* <150 min/week of moderate to vigorous physical activity, *Active*  ≥150 min/week of moderate to vigorous physical activity ^a^ report as sedentary but do not specifically measure moderate to vigorous physical activity, *F* female, *M* male, *NA* no assistance, *LA* low assistance, *HA* high assistance, *CB* conventional bike

Rest periods between conditions ranged from 2-min to 1 month and distance ridden from 3.54 to 27 km. Nine studies were conducted in a natural setting with topography ranging from flat to elevations between 33.5 and 260 m. Four studies specifically examined the impact of topography on physiological outcomes by separating rides into different topographical sections (Additional file 3). Four studies required participants to stop and go during rides to simulate typical riding conditions [20, 26] or delivering mail [24, 25]. In seven studies participants were instructed to ride at a self-selected pace.

#### Longitudinal studies

Six studies examined the longitudinal impact of e-cycling, using a variety of study designs (Table 1). All studies were conducted in high income countries including Belgium, Switzerland, Norway, UK (*n* = 2) and the USA. Sample sizes ranged from 20 to 32, with a total of 153 participants. Most participants were between 30 and 50 years of age. Four studies recruited physically inactive individuals [13, 29–31]. One study included individuals with type 2 diabetes [32] and for one study the health status of individuals was unclear [33]. Interventions ranged from 4-weeks to 8-months in length. One study included published data from mid-point of the intervention, but not post intervention [33]. Three studies provided participants with guidelines on minimum riding requirements, all of which specified riding the e-bike for commuting purposes at least three times per week [13, 29, 30].

### Physical activity intensity

Studies reported a range of outcomes related to physical activity intensity. Given the heterogeneity between studies regarding route length and topography, mean values and/or percent of maximum values during conditions are reported to enable comparison between studies. Physiological outcomes reported within the manuscript include oxygen uptake, metabolic equivalents,^1^ energy expenditure per minute, heart rate and power output (Table 2). Additional outcomes are reported in Additional file 4 and Additional file 5.

**Table 2:** Physical activity intensity outcomes of interest measured during rides^*^

Study	Outcomes	Results; mean (SD)				
		E-bike	Comparison 1	Comparison 2	Comparison 3	Significance testing, *p value*
Bernsten, 2017 [22] ^a^	*(Median, IQR)*	E-bike	CB			
	Percentage VO_2max_	51 (27)	58 (28)			NC
	Measured METs	8.5 (3.1)	10.9 (2.7)			NC
	Estimated METs	6.9 (2.1)	8.4 (1.8)			NC
Cooper, 2018 [32]		E-bike	Walking			
	Mean HR	125.2 (18.1)	107.6 (15.8)			NC
	Men	121.2 (17.2)	103.2 (14.1)			NC
	Women	132.6 (18.9)	116.5 (16.9)			NC
	Percentage HR max	74.7	64.3			NC
Gojanovic, 2011 [18]		E-bike HA	E-bike LA	CB	Walking	
	Mean absolute VO_2peak_	1.50 (.038)	1.79 (0.46)	2.00 (0.44)	1.6 (0.34)	< 0.001 overall, <.05, all comparisons except HA vs. Walk (>.05)
	Percentage VO_2peak_	54.9 (11)	65.7 (8.1)	72.8 (6.4)	59 (9.1)	< 0.001 overall, <.05, all comparisons except HA vs. Walk (>.05)
	Mean estimated METs	6.1 (1.4)	7.3 (1.0)	8.2 (1.3)	6.5 (0.8)	< 0.001 overall, <.05, all comparisons except HA vs. Walk (>.05)
	Mean HR	138.4 (18)	149 (17.7)	157.0 (11.2)	132.7 (17.4)	< 0.001 overall, <.05, all comparisons except HA vs. Walk (>.05)
	Percentage HR max	74.5 (8.7)	80.3 (8.7)	84.6 (5.2)	71.5 (9.2)	< 0.001 overall, <.05, all comparisons except HA vs. Walk (>.05)
Hansen, 2017 [21]		E-bike HA	E-Bike LA	CB		
	Mean absolute VO_2_	1.72 (0.54)	1.89 (0.62)	1.85 (0.52)		.02 overall, .04 LA vs. HA, > .05 CB vs. LA, CB vs. HA
	Percentage VO_2peak_	68 (7.1)	74 (6.2)	73 (4.6)		.01 overall, .03 LA vs. HA, > .05 CB vs. LA, CB vs. HA
	Mean estimated METs	6 (1.8)	6.6 (2)	6.4 (1.6)		.02 overall; .027 HA vs. LA; >.05, CB vs LA, CB vs. HA
Hochsmann, 2017 [30]	*(Median, IQR)*	E-bike	CB			
	Percentage HR max^+^	74.9 (67.4, 82.8)	73.3 (67.7, 78.2)			NC
Langford, 2017 [23] ^a,c^		E-bike	CB	Walking		
	Mean relative VO_2_	16.95 (5.17)	19.32 (5.47)	15.12 (5.35)		NC
	Mean relative EE per minute	0.08 (0.03)	0.10 (0.02)	0.07 (0.03)		NC
	Mean estimated METs	5.1	5.8	4.5		NC
	Mean HR	121.35 (17.04)	127.45 (18.17)	115.25 (14.41)		NC
	Mean power output	63.28 (22.89)	73.13 (35.79)	NA		NC
La Salle, 2017 [26] ^a^		E-bike	CB			
	Mean absolute VO_2_	2.3 (0.1)	2.5 (0.1)			.45
	Percentage VO_2max_	66.4 (2.6)	68 (2.8)			NR
	Mean estimated METs	8.3 (0.5)	8.5 (0.6)			.65
	Mean HR	147 (5)	149 (5)			.064
	Percentage HR max	79.1 (2.4)	80.4 (2.6)			NR
	Mean power output	115 (11)	128 (11)			.38
Louis, 2012 [27] ^b^	*Trained*	E-bike HA	E-bike LA	E-bike NA		
	Mean relative VO_2_	14.7 (2.0)	19.5 (2.4)	22.9 (2.2)		< .05, all comparisons
	Mean estimated METs	4.2 (0.6)	5.6 (0.7)	6.5 (0.6)		< .05, all comparisons
	Mean absolute EE per minute	5.1 (0.8)	7.6 (0.8)	7.8 (0.5)		< .05, all comparisons
	Mean HR	77.7 (11)	89.4 (10.2)	92.8 (11.6)		< .05, all comparisons
	Mean power output	47.3 (9.1)	83.6 (4.0)	104.2 (4.2)		< .05, all comparisons
	*Untrained*	E-bike HA	E-bike LA	E-bike NA		
	Mean relative VO_2_	15.0 (2.0)	21.7 (4.2)	23.4 (3.6)		< .05, all comparisons
	Mean estimated METs	4.3(0.6)	6.2 (1.2)	6.7 (1.0)		< .05, all comparisons
	Mean absolute EE per minute	4.9 (0.8)	6.7 (0.8)	7.5 (0.9)		< .05, all comparisons
	Mean HR	96.8 (16.8)	116.8 (21.7)	116.7 (16.2)		< .05, all comparisons
	Mean power output	40.0 (7.1)	79.8 (4.8)	99.9 (6.9)		< .05, all comparisons
Meyer 2014 [28] ^a^		E-bike	E-bike NA			
	Mean HR	94.71	131.31			NC
Peterman, 2016 [13]		E-bike				
	Mean estimate METs	4.9 (1.2				
	Mean absolute EE per minute	6.5 (1.9)				
	Percentage HR max	72.1 (5.4)				
Simons, 2009 [20]		E-bike HA	E-bike LA	E-bike NA		
	Mean estimated METs	5.2 (1.4)	5.7 (1.2)	6.1 (1.6)		<.05 HA and NA, >.05 HA vs. LA, LA vs. NA
	Mean HR	112.4 (22.9)	116.2 (22.4)	123.8 (23.2)		<.05 NA vs. HA; NA vs. LA, >.05 HA vs. LA
	Percentage HR max	6 7.1 (14.1)	69.3 (13.5)	73.9 (14.5)		<.05 NA vs. HA; NA vs. LA, >.05 HA vs. LA
	Mean absolute power	94.2 (29.2)	101.8 (24.8)	118.2 (30.9)		<.05 All comparisons
Sperlich, 2012 [19] ^a^		E-bike	CB			
	Mean relative VO_2_	18 (3.8)	25.5 (4.8)			<.05, ES = 1.73
	Mean absolute VO_2_	1.33 (0.35)	1.77 (0.43)			< .05, ES = 1.12
	Mean estimated METs	5.2 (1.7)	7.1 (1.4)			<.05, ES = 1.22
	Mean HR	105 (20)	133 (19)			<.05, ES = 1.53
	Mean absolute power	363 (23)	415 (28)			<.05, ES = 2.02
Theurel, 2011 [24]		E-bike	CB			
	Mean absolute EE per minute	5.6 (1.3)	5.9 (1.8)			NR
	Mean HR	NR	NR			.02, 3% lower with e-bike
Theurel, 2012 [25]		E-bike	CB			
	Mean relative VO_2_	29 (5)	37 (5)			< .001
	Mean HR	136 (23)	167 (17)			<.001

^*^Given the difference in the cycle routes conducted mean values or percentage of maximum for outcomes related to physical activity intensity are reported (e.g., Mean VO_2peak_, mean heart rate, mean energy expenditure). For additional physical activity related outcomes reported in the studies see Additional file 4

^+^reported for only a subsample of the group (*n* = 5 e-bikes, *n* = 4 conventional bike)

*EE* energy expenditure, *HR* heart rate, *METs* metabolic equivalent, *VO*_2_ volume of oxygen, *VO*_2_ oxygen intake value; *VO*_2max_ highest oxygen intake value attainable for an individual, *VO*_2peak_ the highest oxygen intake value obtained on a specific test, *CB* conventional bike, *HA* high assistance, *LA* low assistance, *NA* no assistance

*ES* effect size measured as Cohen’s d, *NC* not conducted, *NR* not reported

*Relative VO*_*2*_*, VO*_*2max*_ and *VO*_*2peak*_ measured as ml/min/kg; *Absolute VO*_*2*_*, VO*_*2max*_ and *VO*_*2peak*_ measured in l/min; *Mean absolute energy expenditure* measured in kcal/min; *Mean relative energy expenditure* measured in kcal/kg/min; *Mean heart rate* measured in beats per minute (bpm); *Mean power output* measured in Watts, *Estimated METs* measured using assumption that resting energy expenditure (i.e.,1 MET) = 3.5 ml/kg/min; *Measured METs* measured through assessed individual resting energy expenditure

^a^Results are reported to total cycle routes. Studies separated results for different route topography. See Additional file 3 for details on different cycling topography; ^b^ Participants completed same activity at three different speeds, self-selected speed reported; ^c^ Total sample analyses not conducted, see Additional file 3 for analyses between ride segments

#### Oxygen uptake

Eight studies reported oxygen uptake [18, 19, 21–23, 25–27]. Riding an e-bike led to a relative mean oxygen uptake of 14.7 to 29 ml/min/kg or 51 to 74% of maximum oxygen uptake. E-cycling required lower oxygen uptake than conventional cycling (19.3 to 37 ml/min/kg) or e-cycling with no assistance (22.9 to 23.4 ml/min/kg), with statistically significant differences reported in four studies, one of which reported an effect size of 1.73 [19]. Walking elicited lower oxygen uptake compared to self-selected e-cycling [23] and e-cycling on low assist [18].

#### Metabolic equivalents (METs)

Nine studies reported mean estimated METs while riding an e-bike at a self-selected intensity [13, 18–23, 26, 27], which ranged from 4.9 to 8.3 METs. Overall, e-cycling led to a lower mean MET score than conventional cycling or e-cycling without assistance. However, the significance of the difference is inconclusive. One study reported a difference in mean METs between walking and e-cycling only during uphill sections [23], while another study reported no difference between walking and e-cycling over varied terrain [18].

#### Energy expenditure per minute

Four studies assessed energy expenditure per minute [13, 23, 24, 27]. On an indoor trainer, energy expenditure per minute was lower on an e-bike with assistance (high or low) compared to an e-bike without assistance in physically active adults [27]. In outdoor trials two studies reported no difference in energy expenditure per minute between e-cycling and conventional cycling, though mean values were consistently lower for e-cycling [23, 24]. Absolute energy expenditure per minute while riding an e-bike ranged from 4.9 to 6.5 kcal/min.

#### Heart rate

Twelve studies reported heart rate while e-cycling [13, 18–20, 23–28, 30, 32]. During e-cycling the percentage of maximum heart rate ranged from 67.1 to 79.1. Overall, mean heart rate while riding an e-bike was lower than riding a conventional bike or an e-bike with no assistance. Heart rate showed a trend towards being lower while walking compared to e-cycling [18, 23, 32].

#### Power output

Five studies assessed power output during conditions [19, 20, 23, 26, 27]. Mean power output was lower while riding an e-bike compared to a conventional bike or e-cycling with no assistance. Riding an e-bike on high assistance compared to low assistance led to significantly lower power outputs.

Overall, e-cycling was performed at a moderate intensity, but the intensity was lower than during conventional cycling. Most studies reported significant differences in the associated outcomes between e-cycling and conventional cycling. However, one study found no differences in physiological markers of intensity between e-cycling and conventional cycling [26]. While the evidence is limited, e-cycling appears to be performed at a greater intensity than walking.

### Impact of topography

Five studies directly compared the impact of e-cycling in varying topographies (Additional file 3). The energy cost during e-cycling and conventional cycling uphill ranged from 5.2 to 6.8 and 7.2 to 8.5 METs respectively. This difference was statistically significant in the three studies that conducted hypothesis testing. Examination of means and medians suggested that energy expenditure (METs) during downhill and flat sections were lower while e-cycling compared to conventional cycling, but that this difference in energy cost was less distinct than during uphill sections. Across all studies, greater elevation gains in routes led to higher energy cost for both e-cycling and conventional cycling compared to flat routes or those conducted indoors. Differences in heart rate between e-cycling and conventional cycling appear to be greater during uphill sections, except for one study [19] that reported similar differences in heart rate between cycling conditions across all topographies.

### Physical fitness

A pilot randomized control trial of physically inactive individuals reported an increase in peak oxygen uptake (VO_2peak_) of 10% following 4-weeks of e-cycling compared to a 6% increase following 4-weeks of conventional cycling [30] (Table 3). In a similar population, using a single-group quasi-experimental design, one study reported an 8% increase in VO_2peak_ following 4-weeks of e-cyling [13] and another reported a 7.7% increase in VO_2peak_ following 3-months of e-cycling [31]. When separated into low and high fitness groups a significant increase in VO_2peak_ was reported only in individuals with low levels of fitness, with a 9.6% increase compared to a 1.5% increase in high fitness individuals [31]. Gender differences were reported in one study following 6-weeks of e-cycling with a 2 and 7% increase in VO_2peak_ in physically inactive men and women respectively [29]. Gender differences were also reported in maximum power output with women reporting lower increases in maximum power than men following a 6-week and 5-month intervention [29, 32].

**Table 3:** Results of longitudinal intervention studies

Study	Outcomes	Results, mean, SD (*95% CI)*				
		Intervention		Control		Significance, *p-value*
		Pre	Post	Pre	Post	
		E-bike				
Cooper, 2018 [32]	Max absolute power	157.5 (55.7)	174.3 (70.8)			NC
	Men	182.1 (51.5)	206.2 (64.9)			NC
	Women	118.9 (38.9)	124.3 (49.0)			NC
		E-bike		NE		*Within groups*
De Geus, 2013 [29]	Absolute VO_2peak_					
	Men	2.56 (0.35)	2.61 (0.38)	2.62 (0.46)	2.56 (0.35)	>.0.025 E-bike, NE
	Women	1.94 (0.37)	2.07 (0.41)	1.91 (0.35)	1.94 (0.37)	>.0.025 E-bike, NE
	Relative VO_2peak_					
	Men	30.2 (4.3)	30.7 (5.6)	30.8 (4.9)	30.2 (4.3)	>.0.025 E-bike, NE
	Women	30.0 (6.0)	32.3 (6.5)	29.4 (5.1)	30.0 (6.0)	>.0.025 E-bike, NE
	Absolute max power					
	Men	169.5 (19.9)	192.1 (28.7)	173.8 (27.1)	169.5 (19.9)	<.0.025 E-bike, >.0.025 NE
	Women	130.9 (21.6)	145.9 (24.8)	131.1 (21.7)	130.9 (21.6)	<.0.025 E-bike, >.0.025 NE
	Relative max power					
	Men	2.00 (0.28)	2.30 (0.40)	2.05 (0.35)	2.00 (0.28)	<.0.025 E-bike, >.0.025 NE
	Women	2.03 (0.41)	2.30 (0.55)	2.04 (0.43)	2.03 (0.41)	<.0.025 E-bike, >.0.025 NE
		E-bike		CB		
Hochsmann, 2017 [30]	Relative VO_2peak_	35.7 (5.8)	39.3 (8.3)	36.4 (7.3)	38.6 (6.2)	0.327, 1.4 (− 1.4–4.1)^+^
	Relative power output	2.9 (0.6)	3.2 (0.6)	3 (0.5)	3.3 (0.5)	0.995, 0.0 (− 0.1–0.1)^+^
	Resting HR	64.7 (6.5)	65.1 (7.6)	68.8 (8.8)	65.5 (10.6)	0.505, 2.0 (−4.2–8.2) ^+^
	HR at 100 W max text	113.4 (9.2)	111.5 (7.7)	113.4 (15.9)	109.2 (14.2)	0.219, 2.4 (− 1.5–6.2) ^+^
	SBP at rest	125.9 (13.8)	124.1 (11.3)	127.3 (10.6)	123.1 (12.4)	0.538, 2.0 (−4.5–8.5) ^+^
	DBP at rest	82.4 (8.5)	82.1 (8.2)	87.7 (8)	84.5 (8.8)	0.625, 1.2 (−3.9–6.3) ^+^
	SBP @ 100 W	174.1 (22.9)	160.3 (21.2)	160.8 (20)	150.4 (18.5)	0.93, −0.4 (−9.4–8.7) ^+^
	DBP @ 100 W	86.2 (8.3)	81.9 (6.5)	88 (7.1)	84 (8.1)	0.709, −1.1 (−7.5–5.2) ^+^
		E-bike				
Malnes, 2016 [31]	Relative VO_2peak_	34.1 *(31.6, 36.7)*	36.5 *(34.4, 38.6)*			<.001
	Relative VO_2peak,_ % gain		7.7 *(4.3, 11.1)*			
	High Fitness		1.5 (−5.6, 8.6)			0.626
	Low Fitness		9.6 *(5.9, 13.3)*			<.05
	Peak HR	181 *(175, 187)*	180 *(174, 186)*			0.429
		E-bike commute		Passive commute		
Page, 2017 [33]	QOL (baseline and week 8)	38.00 (3.86)	39.67 (4.47)	29.63 (6.57)	35.71 (5.59)	>.05 E-bike, Passive commute
	OQL (week 4)		38.84 (4.16)		32.67 (6.08)	<.01, ES = 0.28
		E-bike				
Peterman, 2016 [13]	Absolute VO_2max_	2.21 (0.48)	2.39 (0.52)			<.05
	MVPA	28.1 (17.5)	29.0 (20.2)			>.05
	MVPA10+	11.7 (14.3)	13.0 (15.2)			>.05
	Absolute max power	165.1 (37.1)	189.3 (38.3)			<.05
	Fasting glucose	4.99 (0.52)	5.02 (0.47)			>.05
	2 h post plasma glucose	5.53 (1.18)	5.03 (0.91)			<.05
	HOMA	2.46 (0.95)	2.55 (0.82)			>.05
	Total cholesterol	3.90 (0.87)	3.92 (0.79)			>.05
	LDL	2.33 (0.8)	2.34 (0.71)			>.05
	HDL	1.21 (0.24)	1.18 (0.22)			>.05
	Triglycerides	0.95 (0.42)	0.91 (0.27)			>.05
	MAP	84.6 (10.5)	83.2 (9.4)			>.05
	SBP	110.0 (12.4)	109.1 (10.9)			>.05
	DBP	67.7 (8.8)	67.0 (8.0)			>.05

^+^difference between groups, 95% CI, ES = effect size

*Distance* (total and weekly) measured in kilometres; *Duration* (total and weekly) measured in minutes

*NE* no activity, *CB* conventional bike

*SBP* systolic blood pressure, *DBP* diastolic blood pressure, *MAP* mean arterial blood pressure, *QOL* quality of life, *LDL* low density lipo-protein, *HDL* high density lipo-protein, *HOMA* measure of insulin sensitivity using homeostatic model assessment, *MVPA* moderate to vigorous physical activity, *MVPA*10+ moderate to vigorous physical activity of bout of 10-min or greater, *W* watts

VO_2max_ = highest oxygen value attainable for an individual, VO_2peak_ = the highest oxygen intake value obtained on a specific test

*Relative VO*_*2max*_ and *VO*_*2peak*_ measured as ml/min/kg; *Absolute VO*_*2max*_ and *VO*_*2peak*_ measured in l/min *Mean energy expenditure* measured in kcal/min; *Mean heart rate or peak heart rate* measured in beats per minute (bpm); *Mean absolute max power* measured in Watts, *Mean relative power* measured in watts/kg*; glucose, cholesterol, LDL, HDL, Triglycerides* measured in mmol/L; *blood pressure* measured in millimeter of mercury (mmHg), *MVPA* and *MVPA10+* measured in minutes per day

### Health outcomes

Three studies examined the impact of e-cycling on health outcomes beyond fitness (Table 3), for which the outcomes assessed were heterogeneous. After 4-weeks of e-cycling there were no changes in systolic or diastolic blood pressure at rest [13, 30]. There was no evidence of a difference in blood pressure whilst cycling between conventional cycling and e-cycling [30]. Peterman and colleagues [13] reported no changes in insulin resistance or lipid profiles following 4-weeks of e-cycling. However, a significant reduction in 2-h post plasma glucose concentration was reported. No changes were reported in the one study examining quality of life following 8 weeks of e-cycling [33].

### Quality assessment and quality of the evidence

The global rating of acute studies yielded six moderate and five weak ratings according to the EPHPP tool (Table 4). Ten studies were rated as weak for representativeness of the target population, often due to a failure to report how participants were recruited. Methods of assessment were rated as strong. The repeated nature of conditions ensured the control of confounders, therefore yielding a strong rating. Overall there was moderate evidence that e-cycling could lead to physical activity at an intensity associated with beneficial health outcomes [14]. A global rating of strong was given to one longitudinal study, moderate was given to four studies and weak to one study. There was moderate evidence that e-cycling could lead to increased fitness. The evidence related to the impact of e-cycling on additional health outcomes was inconclusive.

**Table 4:** Quality assessment of included studies according to the Effective Public Health Practice Project tool

Study	Component rating						Global rating^a^
	Selection Bias	Design	Confounders	Blinding	Methods	Drop-outs	
Acute studies							
Bernsten [22]	Weak	Strong	Strong	Weak	Strong	Strong	Moderate
Gojanovic [18]	Weak	Moderate	Strong	Weak	Strong	Strong	Moderate
Hansen [21]	Moderate	Strong	Strong	Weak	Strong	Strong	Moderate
Langford [23]	Weak	Moderate	Strong	Weak	Strong	Moderate	Moderate
La Salle [26]	Weak	Strong	Strong	Weak	Strong	Strong	Moderate
Louis [27]	Weak	Strong	Strong	Weak	Strong	Weak	Weak
Meyer [28]	Weak	Weak	Strong	Weak	Strong	Weak	Weak
Simons [20]	Weak	Moderate	Strong	Weak	Strong	Strong	Moderate
Sperlich	Weak	Strong	Strong	Weak	Strong	Weak	Weak
Theurel, 2011 [24]	Weak	Weak	Strong	Weak	Strong	Weak	Weak
Theurel, 2012 [25]	Weak	Weak	Strong	Weak	Strong	Weak	Weak
Longitudinal studies							
Cooper [32]	Moderate	Moderate	Strong	Weak	Strong	Moderate	Moderate
De Geus [29]	Weak	Moderate	Strong	Weak	Strong	Moderate	Moderate
Hochsmann [30]	Moderate	Strong	Strong	Weak	Strong	Strong	Strong
Malnes [31]	Weak	Moderate	Strong	Weak	Strong	Strong	Moderate
Page [33]	Moderate	Weak	Weak	Weak	Strong	Weak	Weak
Peterman [13]	Weak	Moderate	Strong	Weak	Strong	Moderate	Moderate

^a^Strong = no weak component rating; moderate = one weak component rating; weak = two or more weak component ratings

Note: blinding was not included in the overall global rating calculation

## Discussion

The aim of the current review was to assess the intensity of physical activity when riding an e-bike, and to examine the physiological and psychological outcomes associated with e-cycling. Where possible these outcomes were compared to traditional methods of active travel (i.e., walking and cycling). Eleven acute and six longitudinal studies were identified. There was moderate evidence that e-cycling provides moderate intensity physical activity in both physically active and inactive individuals. Furthermore, there was moderate evidence that e-cycling positively impacted cardiorespiratory fitness in physically inactive individuals. The impact of e-cycling on health outcomes beyond physical fitness was inconclusive given the sparsity of current research.

### Quality of the evidence

The quality of all studies, bar one [30], was weak to moderate. These ratings should be viewed with caution as the purpose of physiological studies, such as the acute experiments reported here, is to explore a specific event in a controlled environment with less focus on obtaining representative samples. As such, many studies did not report how participants were recruited, leading to a weak rating for the selection bias component of the assessment. Study design, control of confounders and methods of assessment are often considered more crucial in these designs, all of which were strong in the acute studies reported here. Furthermore, while blinding is often unachievable in physical activity interventions, the use of objective methodology limits the impact of research bias on the outcomes.

Regarding longitudinal studies, methods of data collection were consistently strong, but with large variation in representativeness, design and reporting of withdrawals and dropouts. Confounders were considered in the context of differences between groups and were therefore rated as strong if studies used a single-group design. One pilot randomized control trial was conducted and was rated as strong [30]. Overall, there was a lack of high-quality longitudinal intervention-based research including pre-post measures examining the impact of e-cycling on physiological and psychological health outcomes.

### The impact of e-cycling on physical activity intensity

To accrue health benefits, The American College of Sports Medicine recommend healthy adults engage in moderate-to-vigorous physical activity for 150-min per week [14]. Moderate intensity activity is classified as three to six metabolic equivalents (METs) and vigorous intensity activity at six METs or above. The current review suggests that e-cycling, even while using a high assistance mode, provides physical activity of at least moderate intensity on a variety of terrain, including downhill. Furthermore, e-cycling can elicit vigorous activity during uphill riding [18] and during rides with highly varied terrain [18, 26]. Interestingly, Bernsten and colleagues [22] reported that mean *estimated* METs were lower than mean *measured* METs during e-cycling. Estimated METs have been suggested to overestimate resting energy expenditure, thereby underestimating activity energy expenditure [34]. As such, the mean estimated METs reported in this review provide a conservative estimate of exercise intensity.

Relative physiological outcomes further suggest that e-cycling is performed at a moderate intensity with the percent of maximum heart rate ranging from 67.1 to 79.1 and the percent of VO_2peak/max_ ranging from 51 to 75. These values exceed the hypothesised minimum intensity thresholds required for improvements in cardiorespiratory fitness in healthy adults [14, 35, 36].

### E-cycling vs. traditional active transportation

Three studies compared e-cycling to walking [18, 23, 32] of which one compared the two modes on the same route [23]. In this study walking led to lower oxygen uptake than e-cycling across all topographies, though significant MET differences were only reported during uphill sections, with e-cycling expending more energy than walking. The few studies conducted suggest e-cycling is performed at a higher intensity than walking, however, more studies are needed to confirm these trends.

In relation to conventional cycling, this review suggests that e-cycling elicits lower physiological markers of intensity than conventional cycling, however the strength of this finding depends on the physiological assessment measure and route topography. Overall, mean percent of VO_2max/peak_ is similar between conventional cycling and e-cycling ranging from 58 to 74% and 51 to 73% respectively. Studies examining active commuting on conventional bikes have reported similar mean percent of VO_2max_ in healthy adults ranging from 57 to 79% [6, 37]. However, mean relative oxygen uptake is lower during e-cycling compared to conventional cycling or e-cycling without assistance. Similarly, means and medians of estimated METs are consistently higher during conventional cycling or e-cycling without assistance compared to assisted e-cycling, with values ranging from 6.1 to 8.5 and 4.9 to 8.3 respectively, though the significance of the differences varied across studies.

La Salle and colleagues [26] reported similar MET values between e-cycling and conventional cycling. However, the values reported were substantially higher than those reported in other studies, with mean estimated METs of 8.3 and 8.5 for e-cycling and conventional cycling respectively. Participant demographics may have accounted for these differences, since participants were younger and had previous cycling experience. These participants may have had higher aerobic capacity and therefore self-selected a higher intensity activity level at which to complete the conditions. This is likely given that the relative intensity of activity is similar in studies of e-cycling in physically inactive individuals [13, 18–20, 30, 32]. When given the choice to self-select pace and intensity individuals may select a similar physiological intensity across activities regardless of the mechanical assistance, thereby resulting in similar physiological outcomes. In support of this, when individuals were required to maintain a cycling cadence of 60 revolutions per minute throughout a condition, there were significant differences in oxygen uptake and heart rate between e-bikes and conventional bikes [18] compared to studies in which individuals were able to self-selected their intensity [21, 22, 26]. Similarly, when instructed to complete 60-meters of riding in 10-sec for a total of 30-min the reported relative VO_2max_ was 29 ml/min/kg for e-cycling and 37 ml/min/kg for conventional cycling [25]. This suggests that performing the same amount of work requires more effort on a conventional bike than an e-bike, but that human beings reduce the amount of work conducted on a conventional bike, through choosing a slower speed, to account for the increase in expended effort.

In hilly terrain, where there is less opportunity to adjust effort levels to produce comparable intensity levels, the differences between conventional cycling and e-cycling may become more pronounced, with e-cycling requiring lower intensity activity, as found in studies comprised of routes with hilly features [18, 23]. This suggests that e-bikes are less sensitive to environmental factors such as topography. Therefore, physiological measures of intensity are lower on the e-bike than those reported on a conventional bike during uphill riding. The reduced intensity required during uphill riding when using an e-bike is one of the leading arguments for the promotion of e-bikes as an alternative mode of active transportation.

### E-cycling and health

In the current review three studies provided weekly e-cycling goals for physically inactive individuals in the context of active commuting [13, 29, 30]. Two of these studies reported increases in VO_2peak_ and maximum power output following 4-weeks of e-cycling [13, 30]. In contrast de Geus and colleagues [10] reported no changes in VO_2peak_ following a 6-week intervention, though differences in maximum power output were seen. Differences between studies could be due to distance cycled. Specifically, both Hochsmann [30] and Peterman and colleagues [13] reported cycling distances of 70 km and 69.4 km per week respectively, compared to 54.3 km per week reported by de Geus [10]. The two studies reporting significant increases in fitness also described self-selected riding intensities of between 72.1 and 74.9% of maximum heart rate (within the moderate intensity zone [13, 30] with an average of 205 min (±43.3) of e-cycling per week [13]. This suggests that e-cycling can contribute to meeting weekly physical activity guidelines.

Without the provision of e-cycling goals, single group studies with physically inactive individuals reported increases in maximal power output of 7 to 10% over 3–8 months, despite lower average distance travelled than other studies [31, 32]. Fitness benefits were greatest in individuals classified as having low fitness [31], similar to findings with conventional cycling [6]. These results suggest that in the absence of specific goals (i.e., under free living conditions), participants engage in e-cycling and this e-cycling can contribute to improvements in fitness.

Beyond cardiorespiratory fitness, there is a lack of research examining the impact of e-cycling on physiological or psychological health outcomes, limiting our ability to draw conclusions. Peterman and colleagues [13] reported a decrease in 2-h plasma glucose during an oral glucose tolerance test after 4-weeks of e-cycling. This finding is in line with studies that have examined the impact of exercise on 2-h post exercise glucose concentrations in obese individuals [38, 39] but is novel in the context of e-cycling and conventional cycling. In the same study, no other metabolic changes were reported. Similar null effects on metabolic outcomes were reported in two systematic reviews on conventional cycling [37, 40].

### E-cycling for public health?

Overall e-cycling can elicit at least moderate intensity physical activity. However, total energy expenditure when riding an e-bike is lower than when riding a conventional bike or walking over the same distance, given the reduced amount of time taken to complete a ride on an e-bike. Consequently, if e-cycling were to replace journeys made by walking or conventional cycling, individuals would have to ride for longer for comparable weekly energy expenditure. However, e-cycling is associated with lower ratings of perceived exertion than conventional cycling [23, 26], potentially enabling people to ride more frequently or for a longer duration. This possibility is supported by Hendriksen and colleagues [41], who reported that individuals in the Netherlands commuted 50% further with an e-bike than on a conventional bike.

Findings reported here suggest that e-cycling may be suitable for individuals with compromised health. Hansen and colleagues [21] showed that e-cycling elicited moderate intensity activity in older, obese individuals recovering from surgery due to coronary artery disease, while Cooper and colleagues [32] reported that e-cycling was feasible for middle-aged, overweight individuals with type 2 diabetes mellitus.

Overall, while there is a trend towards increased fitness following engagement in e-cycling interventions, more intervention research of a longer duration is required before the long-term impact of e-cycling on health can be determined. Fifty percent of the longitudinal studies in this review were approximately 1-month in length. This may not be enough time to see changes in body composition and some metabolic outcomes. Longer trials with larger samples sizes should be conducted with a focus on including a range of health outcomes in addition to cardiorespiratory fitness. These trials should utilize randomized controlled designs and clearly report their target population, recruitment process and dropouts and/or withdrawals. Interventions should also be conducted in clinical populations where physical activity is compromised. In addition, more research is needed to understand the impact of e-cycling on health based on sex or fitness level.

It is also important to consider the negative outcomes associated with e-cycling when assessing their potential utilization for health promotion. In the USA, e-bike users reported feeling safer riding their e-bike than a conventional bike, stating that the e-bike helped them to avoid crashes due to their stability, powerful brakes and the acceleration to avoid incidents and keep up with traffic. However, riders reported cycling faster on an e-bike than a conventional bike and felt that other road users misjudged their speed leading to potentially dangerous situations [42]. In the Netherlands data suggest that, after controlling for age, gender and amount of cycling, use of an e-bike was associated with an increased risk of being involved in a crash compared to conventional cycling [43]. The severity of these crashes was not significantly different from conventional cycling [43]. More context specific research is required to enable a risk-benefit assessment of engaging specifically in e-cycling. Nevertheless, e-cyclists would be well advised to be appropriately trained and use safety equipment to minimize risk.

### Strengths and limitations

This is the first review to examine the physical activity intensity, cardiorespiratory, metabolic and psychological outcomes associated with e-cycling. This review used two pragmatic tools to assess the quality of studies and to provide an overall rating of the evidence. These tools provided an overall representation of the strength of research evidence related to e-cycling and health. Limitations of this review include the fact that some published studies may not have been identified. However, our systematic and broad search strategy makes this unlikely. It is more likely that we did not identify eligible unpublished studies or those published in an alternative language to English. Sample sizes used in studies were small and sample size calculations were rarely reported. Therefore, caution should be taken when interpreting the statistical significance of evidence. Given the heterogeneity in outcome measurement we were unable to quantify the effects of e-cycling on outcomes of interest using meta-analyses. In addition, focus on quality of life as a psychological outcome may have meant studies examining psychological outcomes such as depression or anxiety were excluded.

## Conclusion

The composite results of the 17 studies included in this novel systematic review provide moderate evidence that e-cycling elicits activity at an intensity high enough to promote some positive health outcomes. E-cycling leads to reduced activity volume and intensity over the same distance compared to conventional cycling. Therefore, e-cycling requires more frequent and longer rides to accrue comparable health benefits. However, given that most individuals travel by car to work [44] e-cycling offers a physically active alternative to the largely sedentary behaviour associated with motorized commuting. Furthermore, longitudinal studies suggest, with moderate confidence, that e-cycling can lead to increases in cardiorespiratory fitness. Longer and higher-quality intervention studies, with transparent reporting, are needed to develop a strong evidence-based understanding of the impact of e-cycling on cardiorespiratory health and to explore the impact of e-cycling on metabolic and psychological outcomes. This will extend the current body of knowledge and provide guidance on public health initiatives to promote e-cycling to improve population health.

## Additional files

Additional file 1: Example search strategy. (DOCX 12 kb)

Additional file 2: Description of overall strength of evidence criteria. (DOCX 12 kb)

Additional file 3: Outcomes of interest by route topography for acute experimental and quasi-experimental studies. (DOCX 23 kb)

Additional file 4: Additional physical activity outcomes measured in acute studies. (DOCX 28 kb)

Additional file 5: Additional physical activity outcomes measured in longitudinal studies. (DOCX 22 kb)

## Footnotes

1 The MET is an expression of energy cost and is calculated from rest where 1 MET is estimated to equal 3.5 ml/kg/min
