# Supplementary material for: Health benefits of electrically-assisted cycling: a systematic review
Source: Int J Behav Nutr Phys Act. 2018 Nov 21;15:116. doi: 10.1186/s12966-018-0751-8 (PMC6249962; doi:10.1186/s12966-018-0751-8)
Supplement: Supplementary file 1 — Example search strategy. (DOCX 12 kb) [file 12966_2018_751_MOESM1_ESM.docx]

**Additional File 1.** *Example search strategy*

**EBIKE MEDLINE, EMBASE AND PSCHINFO**

1. pedelec*.ti,ab.

2. (electric* adj1 (assist* adj1 bicyc*)).ti,ab.

3. (electric* adj1 (assist* adj1 cyc*)).ti,ab.

4. (electric* adj1 (assist* adj1 bike*)).ti,ab.

5. e-bike*.ti,ab.

6. (electric* adj1 bike*).ti,ab.

7. (electric* adj1 bicyc*).ti,ab.

8. (electric* adj1 cyc*).ti,ab.

9. (pedal-assist* adj1 electric* adj1 bike*).ti,ab.

10. (pedal-assist*).ti,ab.

11. (electrically-assist* adj1 bike*).ti,ab.

12. (electrically-assist* adj1 bicyc*).ti,ab.

13 (electrically-assist* adj1 cyc*).ti,ab.

14. (electric adj1 mobil*).ti,ab.

15. 1 or 2 or 3 or 4 or 5 or 6 or 7 or 8 or 9 or 10 or 11 or 12 or 13 or 14

No subject headings
