# Supplementary material for: Health benefits of electrically-assisted cycling: a systematic review
Source: Int J Behav Nutr Phys Act. 2018 Nov 21;15:116. doi: 10.1186/s12966-018-0751-8 (PMC6249962; doi:10.1186/s12966-018-0751-8)
Supplement: Supplementary file 2 — Description of overall strength of evidence criteria. (DOCX 12 kb) [file 12966_2018_751_MOESM2_ESM.docx]

**Additional File 2.** *Description of overall strength of evidence criteria*

**Strong evidence**: 1) at least two RCTs (included randomized cross over studies) of high quality or 2) one RCT of high quality and at least two RCTs of medium quality. Effects must be consistent in both cases.

**Moderate evidence**: 1) one RCT of medium quality and at least one RCT of low quality or 2) one RCT of medium quality and at least one controlled trial (CT) of high quality or 3) at least three CTs of high quality or 4) one CT of high quality and at least three CTs of medium quality. Effects must be consistent in all cases.

**Limited evidence:** 1) more than one RCT of low quality or 2) one CT of medium quality and two CTs of low quality or 3) two CTs of low quality and at least two before-after, cohort or longitudinal studies. Effect must be consistent in all cases.

**Inconclusive evidence:** 1) only one study or 2) multiple before-after, cohort, or longitudinal studies, or 3) contradictory effects.

**No evidence:** more than one study with consistent non-significant effects

**Based on previously utilized method (**De Bourdeaudhuij I, Van Cauwenberghe E, Spittaels H, et al. School-based interventions promoting both physical activity and healthy eating in Europe: a systematic review within the HOPE project. *Obesity Reviews* 2011;12(3):205-16. doi: 10.1111/j.1467-789X.2009.00711.x)
