# Supplementary material for: Health benefits of electrically-assisted cycling: a systematic review
Source: Int J Behav Nutr Phys Act. 2018 Nov 21;15:116. doi: 10.1186/s12966-018-0751-8 (PMC6249962; doi:10.1186/s12966-018-0751-8)
Supplement: Supplementary file 3 — Outcomes of interest by route topography for acute experimental and quasi-experimental studies. (DOCX 23 kb) [file 12966_2018_751_MOESM3_ESM.docx]

**Additional File 3.** Outcomes of interest by route topography for acute experimental and quasi-experimental studies

| **Study** | **Physical activity outcomes of interest measured** | **Results, mean, SD** | | | | | | | | | | | | | | | | | |
| --- | --- | --- | --- | --- | --- | --- | --- | --- | --- | --- | --- | --- | --- | --- | --- | --- | --- | --- | --- |
|  |  | **Route 1: Flat** | | |  | | **Route 2: Hilly** | | |  | |  | |  |  |  | |  | |
|  | *(Median, IQR)* | **E-bike** | **CB** |  | | **E-bike** | | **CB** |  | |  | |  | |  |  | |  | |
| Bernsten, 2017^a^ | Percentage VO_2_max | 52(19) | 55 (12) |  | | 50 (18) | | 60 (22) |  | |  | |  | |  |  | |  | |
|  | Measured METs | 8.5 (3.1) | 10.3 (2.8) |  | | 8.4 (3.2) | | 10.8 (3.1) |  | |  | |  | |  |  | |  | |
|  | Estimated METs | 6.9 (1.9) | 8.1 (2.5) |  | | 6.8 (2.5) | | 8.5 (2.1) |  | |  | |  | |  |  | |  | |
|  |  | **Section 1: 0.885km** | | |  | | **Section 2: 0.885km** | | |  | | **Section 3: 0.885km** | | |  | **Section 4: 0.885km** | | | |
|  |  | **E-bike** | **CB** |  | | **E-bike** | | **CB** |  | | **E-bike** | | **CB** | |  | **E-bike** | | **CB** | |
| La Salle, 2017 | Mean absolute VO_2_ | NR | NR* |  | | NR | | NR* |  | | NR | | NR* | |  | NR | | NR* | |
|  | Percentage VO_2_ max | NR | NR |  | | NR | | NR |  | | NR | | NR | |  | NR | | NR | |
|  | Mean estimated METs | NR | NR* |  | | NR | | NR* |  | | NR | | NR* | |  | NR | | NR* | |
|  | Mean HR | NR | NR |  | | NR | | NR |  | | NR | | NR | |  | NR | | NR* | |
|  | Percentage HR max | NR | NR |  | | NR | | NR |  | | NR | | NR | |  | NR | | NR | |
|  | Mean power output | NR | NR |  | | NR | | NR |  | | NR | | NR | |  | NR | | NR | |
|  |  | **Section 1: Downhill** | | | | | **Section 2: Flat** | | | | | **Section 3: Uphill** | | | | |  | |  |
|  |  | **E-bike** | **CB** | **Walking** | | **E-bike** | | **CB** | **Walking** | | **E-bike** | | **CB** | | **Walking** |  | |  | |
| Langford, 2017 | Mean relative VO_2_ | 13.0  (4.81) | 13.8  (5.18)* | 13.4  (5.25)* | | 15.9  (6.05) | | 18.2  (7.57)* | 14.6 (5.56)* | | 23.2  (5.10) | | 26.6  (4.72)* | | 18.5 (5.42)* |  | |  | |
|  | Mean EE per minute | 0.06  (0.2) | 0.07  (0.03) | 0.07  (0.03) | | 0.08  (0.03) | | 0.09  (0.04) | 0.07 (0.03) | | 0.12  (0.03) | | 0.13  (0.02)* | | 0.09 (0.03)* |  | |  | |
|  | Mean estimated METs | 3.7 | 3.9 | 3.8 | | 4.5 | | 5.2 | 4.1 | | 6.6 | | 7.6* | | 5.3* |  | |  | |
|  | Mean HR | 109.5  (13.1) | 111.3  (12.9) | 109.5  (13.1) | | 118.2  (19.5) | | 121.3  (30.1) | 114.0 (14.7) | | 140.3 (20.5) | | 152.1  (17.0)* | | 126.5 (16.6)* |  | |  | |
|  | Mean power output | 36.3  (18.9) | 52.4  (16.5)* |  | | 62.4  (28.2) | | 93.0  (22.4)* |  | | 98.3  (25.8) | | 117.4  (27.7) | |  |  | |  | |
|  |  | **Section 1: small asphalt uphill** | | | | | **Section 2: long gravel uphill** | | |  | | **Section 3: short uphill on gravel** | | | | | **Section 4: downhill gravel** | | |
|  |  | **E-bike** | **CB** |  | | **E-bike** | | **CB** |  | | **E-bike** | | **CB** | |  | **E-bike** | | **CB** | |
| Meyer, 2014^a^ | Mean HR | 89.37 (3.92) | 115.77 (10.57) |  | | 91.81 (5.60) | | 137.48 (8.13) |  | | 95.34 (3.31) | | 154.24 (9.59) | |  | 102.31 (1.75) | | 117.76 (13.90) | |
|  |  | **Section 1: Uphill** | | |  | | **Section 2: Downhill** | | |  | | **Section 3: Uphill** | | |  | **Section 4: Flat** | | | |
|  |  | **E-bike** | **CB** |  | | **E-bike** | | **CB** |  | | **E-bike** | | **CB** | |  | **E-bike** | | **CB** | |
| Sperlich, 2012 | Mean relative VO_2_ | 18.3 (4.6) | 25.7 (4.8)* |  | | 16.9 (3.2) | | 23.2 (4.6)* |  | | 18.9 (4.3) | | 27.4 (5.3)* | |  | 18.0 (3.3) | | 25.7 (5.3*) | |
|  | Mean absolute VO_2_ | 1340 (373) | 1824 (450)* |  | | 1271 (356) | | 1656 (418)* |  | | 1390 (358) | | 1942 (439)* | |  | 1330 (380) | | 1839 (356)* | |
|  | Mean estimated METs | 5.2 (1.2) | 7.2 (1.5)* |  | | 4.8 (0.9) | | 6.5 (1.3)* |  | | 5.8 (2.8) | | 7.7 (1.6)* | |  | 5.1 (1.2) | | 7.3 (1.2)* | |
|  | Mean HR | 108 (18) | 136 (17)* |  | | 104 (20) | | 133 (21)* |  | | 105 (17) | | 137 (16)* | |  | 100 (20) | | 140 (19)* | |
|  | Mean absolute power | 89 (35) | 105 (49)* |  | | 72 (37) | | 116 (32)* |  | | 84 (27) | | 122 (39)* | |  | 76 (33) | | 120 (33)* | |
| *significantly different from e-biking, ^a^ no significant testing conducted  NR = not reported, EE = energy expenditure, HR = heart rate, METs = metabolic equivalent, VO_2_ = volume of oxygen  *Relative VO_2_, VO_2max_* and *VO_2peak_* measured as ml/min/kg; *Absolute VO_2_, VO_2max_* and *VO_2peak_* measured in l/min *Mean energy expenditure* *per minute* measured in kcal/min;  *Heart rate* measured in beats per minute (bpm); *Mean absolute max power* measured in Watts, *Mean relative power* measured in watts/kg, *Estimated METs* measured using assumption that resting energy expenditure (i.e.,1 MET) = 3.5ml/kg/min; *Measured METs* measured through assessed individual resting energy expenditure | | | | | | | | | | | | | | | | | | | |
