# Supplementary material for: Health benefits of electrically-assisted cycling: a systematic review
Source: Int J Behav Nutr Phys Act. 2018 Nov 21;15:116. doi: 10.1186/s12966-018-0751-8 (PMC6249962; doi:10.1186/s12966-018-0751-8)
Supplement: Supplementary file 4 — Additional physical activity outcomes measured in acute studies. (DOCX 28 kb) [file 12966_2018_751_MOESM4_ESM.docx]

| **Additional File 4.**  Additional physical activity outcomes measured in acute studies | | | | | | |  |
| --- | --- | --- | --- | --- | --- | --- | --- |
| **Study** | **Physical activity outcomes of interest measured*** | **Results, mean, SD** | | | | |  |
|  |  | **E-biking** | **Comparison 1** | **Comparison 2** | **Comparison 3** | **Significance testing, *p value*** |  |
|  | Total Route | **E-bike** | **CB** |  |  |  |  |
| Bernsten, 2017^a^ | Time to completion | 19.9 (3.1) | 25.1 (3.9) |  |  | NC |  |
|  |  | **E-bike HA** | **E-bike LA** | **CB** | **Walking** |  |  |
| Gojanovic, 2011, Switzerland | Time to completion | 18:48 (2:16) | 20:45 (3:12) | 29:36 (1:34) | 22:06 (1:34) | <.001 all comparisons except Walk vs. LA (>0.5) |  |
|  | Absolute VO_2_ peak | 1.99 (0.57) | 2.36 (0.62) | 2.65 (0.62) | 2.22 (0.49) | <.001 overall, individual comparisons NR |  |
|  | MET-minutes | 114.1 (13.7) | 145.8 (22.4) | 252.8 (42.1) | 144.1 (13.7) | <.001 overall, individual comparisons NR |  |
|  | Peak Heart Rate | 157.9 (17.4) | 168.1 (16.2) | 175.6 (9.6) | 149.1 (19.8) | <.001 overall, individual comparisons NR |  |
|  |  | **E-bike HA** | **E-Bike LA** | **CB** |  |  |  |
| Hansen, 2017, Belgium | Time to completion | 31 (4.7) | 35 (5.3) | 37 (6.5) |  | <.001 overall, NA vs. HA, LA vs. HA, .301 NA vs. LA |  |
|  | Estimated total EE | 249 (53) | 301 (57) | 312 (45) |  | >.05 NA vs LA, <.001 NA vs HA; LA vs HA |  |
|  | MET-minutes | 183 (36) | 222 (36) | 230 (24) |  | <.001 overall, NA vs. HA, LA vs. HA, >.05 NA vs. LA |  |
|  | Mean VCO_2_ | 1542 (496) | 1734 (569) | 1742 (531) |  | .003 overall, 1.0 NA vs. LA, .03 NA vs. HA, .03 LA vs HA |  |
|  | Ventilation^*^ | 55 (16.8) | 63.9 (20.9) | 60.7 (16) |  | .01 overall, 018 LA vs. HA, >.05 NA vs. LA, NA vs. HA |  |
|  | Respiratory exchange ratio | 0.9 (0.1) | 0.9 (0.1) | 0.9 (0.1) |  | 0.095 overall |  |
|  |  | **E-bike** | **CB** |  |  |  |  |
| La Salle, 2017 | Time to completion | 12.5 (0.3) | 13.8 (0.3) |  |  | .01 |  |
|  | **Total Route** | **E-bike** | **CB** | **Walking** |  | **mode x segment** |  |
| Langford, 2017 | Time to completion | 15.77 (1.57) | 17.64 (1.45) | 48.87 (3.99) |  | <.001 overall, all segments <.05 e-bike vs CB, e-bike vs walk |  |
|  | Relative total EE | 1.30 (0.37) | 1.71 (0.51) | 3.61 (1.31) |  | <.001 overall, all segments <.05 e-bike vs CB, e-bike vs walk |  |
|  | Mean relative EE per trip | 0.30 (0.08) | 0.39 (0.12) | 0.83 (0.30) |  | Overall NR, <.05 CB vs E-bike, Walk vs. E-bike |  |
|  | Absolute VO_2_ | 0.26 (0.07) | 0.34 (0.10) | 0.73 (0.26) |  | Overall NR, <.05 CB vs E-bike, Walk vs. E-bike |  |
|  | ***Trained*** | **E-bike HA** | **E-bike LA** | **E-bike NA** |  |  |  |
| Louis, 2012, France^b^ | Ventilation | 24.1 (3.5) | 31.7 (3.4) | 35.0 (4.0) |  | <.05 all comparisons |  |
|  | Gross efficiency | 13.8 (1.5) | 18.7 (2.5) | 19.6 (1.2) |  | <.05 NA vs. HA, LA vs HA, >.05 NA vs. LA |  |
|  | ***Untrained*** | **E-bike HA** | **E-bike LA** | **E-bike NA** |  |  |  |
|  | Ventilation | 24.9 (3.6) | 35.1 (4.8) | 37.3 (5.3) |  | < .05 NA vs. HA, LA vs HA, >0.5 NA vs. LA |  |
|  | Gross efficiency | 12.3 (2.2) | 17.1 (2.6) | 19.5 (1.9) |  | < .05 all comparisons |  |
|  |  | **E-bike** | **E-bike NA** |  |  |  |  |
| Meyer, 2014, Germany | Blood lactate | 0.93 (0.13) | 3.65 (1.29) |  |  | NC |  |
|  |  | **E-bike HA** | **E-bike LA** | **E-bike NA** |  |  |  |
| Simons, 2009 | Time to completion | 11:33 (0:58) | 12:45 (1:26) | 13:38 (1:49) |  | < .05 all comparisons |  |
|  | Total absolute EE | 77.5 (20) | 94.3 (14.9) | 108.1 (18.4) |  | < .05 all comparisons |  |
|  | MET-minutes | 60.3 (13.6) | 71.6 (12.2) | 81.4 (15.4) |  | < .05 all comparisons |  |
|  | Maximum Heart Rate | 125.5 (23.2) | 130.5 (23.3) | 135.8 (23.6) |  | < .05 NA vs. HA, > .05 HA vs LA, LA vs. NA |  |
|  | Maximum power output | 253.8 (92.4) | 266.6 (104.2) | 259.1 (79.5) |  | >.05 all comparisons |  |
|  | **Total Route** | **E-bike** | **CB** |  |  |  |  |
| Sperlich, 2012^a^ | MET-minutes | 192 (62) | 291 (57) |  |  | <.05, ES = 1.66 |  |
|  | Oxygen cost of exercise | 16.6 (4.8) | 15.7 (6.8) |  |  | >.05, ES = 0.15 |  |
|  | Respiratory exchange ratio | 0.86 (0.06) | 0.94 (0.10) |  |  | < .05, ES = 0.97 |  |
|  | Breathing frequency | 31 (4) | 33 (5) |  |  | >.05, ES = 0.44 |  |
|  | Ventilation | 37.6 (6.7) | 52.6 (12) |  |  | < .05, ES = 1.54 |  |
|  | Blood lactate |  |  |  |  |  |  |
|  | Pre | 0.6 (0.1) | 0.6 (0.1) |  |  |  |  |
|  | Post | 1.4 (0.8) | 3.9 (3.0) |  |  | <.05, ES = 1.13 |  |
|  | EMG amplitude |  |  |  |  |  |  |
|  | Biceps femoris | 16.3 (15.7) | 31.8 (23.3) |  |  | < .05, ES = 0.78 |  |
|  | Vastus lateralis | 28.9 (26.8) | 43.1 (40) |  |  | < .05, ES = 0.41 |  |
|  | Vastus medialis | 35 (32.2) | 54.8 (50.2) |  |  | <.05, ES = 0.46 |  |
|  | Gastrocnemius medialis | 27.3 (15.4) | 37.6 (32.6) |  |  | < .05, ES = 0.36 |  |
|  |  | **E-bike** | **CB** |  |  |  |  |
| Theurel, 2011, France | Time to completion | 160 (27) | 166 (40) |  |  | .37 |  |
|  | Absolute total EE | 923 (324) | 933 (267) |  |  | .91 |  |
|  | Heart rate greater than 5% total work time | NR | NR |  |  | <.001, 17% lower when using e-bike compared to CB |  |
|  | Time with EE greater than 6METs | 23 (23) | 28 (25) |  |  | .46 |  |
|  |  | **E-bike** | **CB** |  |  |  |  |
| Theurel, 2012, France | Respiratory exchange ratio | 0.86 (0.03) | 0.9 (0.05) |  |  | <.05 |  |
|  | Mean HR | 136 (23) | 167 (17) |  |  | <.001 |  |
|  | EMG amplitude |  |  |  |  |  |  |
|  | Rectus femoris | 49 (4) | 69 (12) |  |  | <.001 |  |
|  | Vastus lateralis | 50 (4) | 64(11) |  |  | <.001 |  |
|  | Gastrocnemius medialis | 47 (26) | 66 (18) |  |  | <.001 |  |
| T = trained, UT = untrained, NC = not conducted, ES = effect size  EE = energy expenditure, HR = heart rate; VO_2peak_ = the highest oxygen intake value obtained on a specific test; VCO_2_ = carbon dioxide output; EMG = electromyography  *Time to completion* measured in minutes; *Total energy expenditure* measured in kcal; *ventilation and breathing frequency* measured in l/min (**ventilation* measured in ml/min); *Heart rate* measured in beats per minute (bpm);  *Relative VO_2_, VO_2max_* and *VO_2peak_* measured as ml/min/kg; *Absolute VO_2_, VO_2max_* and *VO_2peak_* measured in l/min; *oxygen cost of exercise* measured as ml/min/Watts VCO_2_ measured in ml/min; *EMG amplitude* measured in µV; respiratory exchange ratio = ratio between amount of carbon dioxide produced and oxygen used; *Power output* measured in Watts; *Total relative total* *EE* measured as kcal/kg; *Relative EE per trip* measured as kcal/kg/km; *Total absolute EE* measured as kcal; *Estimated total EE* measured as METscore/kg/cycling hrs; *MET-minutes* measured as METscore/mins; *Working time with EE greater than 6METs* measured as minutes; *Gross efficiency* measured as a percent (Gross efficiency is the percentage ratio of external work achieved compared to total energy expended); *Blood lactate* measured in mmol/l | | | | | | | |
