# Supplementary material for: Health benefits of electrically-assisted cycling: a systematic review
Source: Int J Behav Nutr Phys Act. 2018 Nov 21;15:116. doi: 10.1186/s12966-018-0751-8 (PMC6249962; doi:10.1186/s12966-018-0751-8)
Supplement: Supplementary file 5 — Additional physical activity outcomes measured in longitudinal studies. (DOCX 22 kb) [file 12966_2018_751_MOESM5_ESM.docx]

| **Additional File 5.**  Additional physical activity outcomes measured in longitudinal studies | | | | | | | | | | | | | | | |
| --- | --- | --- | --- | --- | --- | --- | --- | --- | --- | --- | --- | --- | --- | --- | --- |
| **Study** | **Outcomes** | **Results, mean, SD, (*95% CI)*** | | | | | | | | | | | | | |
|  |  | **Intervention** | | | | | **Control** | | | | |  | | | |
|  |  | **During intervention** | | **Pre** | **Post** | **During Intervention** | | **Pre** | | ***Post*** | | | **Significant, p-value** | |  |
|  | *Median (IQR)* | **E-bike** | | | | | **Walking** | | | | | | |  | |
| Cooper, 2018 | Total distance | 383.5  (103, 738.3) | |  |  |  | |  | |  | | | NC | |  |
|  | **Men** | 456 (379, 1395) | |  |  |  | |  | |  | | |  | |  |
|  | **Women** | 111 (73, 252) | |  |  |  | |  | |  | | |  | |  |
|  | Weekly distance | 21.4  (5.5, 37.7) | |  |  |  | |  | |  | | | NC | |  |
|  | **Men** | 23.1 (21.3, 72.9) | |  |  |  | |  | |  | | |  | |  |
|  | **Women** | 6.2 (5.5, 14.9) | |  |  |  | |  | |  | | |  | |  |
|  | Mean number journeys per week testing week | 4.5 (3.3) | |  |  | 1 (1.1) | |  | |  | | |  | |  |
|  | Mean ride distance on testing week | 7.5 (4.2) | |  |  | 1.0 (1.1) | |  | |  | | | NA | |  |
|  | Mean ride duration testing week | 26.6 (12.6) | |  |  | 16 (17.2) | |  | |  | | | NA | |  |
|  |  | **E-bike** | | | | | **No activity** | | | | | | |  | |
| De Geus, 2013 | Average distance per day* | | 15.5 (4.6) |  |  |  | | | | | | |  | |  |
|  | Total distance* | | | | | | | | | | | | | | |
|  | **Men** | 405.1 (156) | |  |  |  | |  | |  | | |  | |  |
|  | **Women** | 246 (116.3) | |  |  |  | |  | |  | | | 0.019 | |  |
|  | Mean ride frequency per week | | |  |  |  | |  | |  | | |  | |  |
|  | **Men** | 4.1 (1.7) | |  |  |  | |  | |  | | |  | |  |
|  | **Women** | 2.9 (1.0 | |  |  |  | |  | |  | | | 0.065 | |  |
|  | Absolute power at blood lactate 2mmol/l | | | | | | | | | | | | | | |
|  | **Men** |  | | 94.6 (28.2) | 121.6 (35.4) |  | | 96.4 (43.1) | | 94.6 (28.2) | | | .0604, >.0.025 No activity | |  |
|  | **Women** |  | | 80.7 (22.3) | 106.1 (29.7) |  | | 72.7 (20.9) | | 80.7 (22.3) | | | 0.001 e-bike, >.0.025 No activity | |  |
|  | Absolute power at blood lactate 4mmol/l | | | | | | | | | | | | | | |
|  | **Men** |  | | 151.1 (27.1) | 174.0 (30.7) |  | | 149.1 (35.3) | | 151.1 (27.1) | | | <.0.025 e-bike  >.0.025 No activity | |  |
|  | **Women** |  | | 117.2 (19.6) | 135.1 (22.6) |  | | 113.4 (17.1) | | 117.2 (19.6) | | | <.0.025 e-bike  >.0.025 No activity | |  |
|  |  | **E-bike** | | | | **CB** | | | | | | |  | |  |
| Hochsmann, 2017 | Total distance* | 280.8 (101.6) | |  |  | 289.6 (131.5) | |  | |  | | | 0.843 | |  |
|  | Additional PA* | 67.1 (66.8) | |  |  | 87.9 (78.7) | |  | |  | | | 0.452 | |  |
|  |  | **E-bike** | | | | |  | |  | |  | |  | | |
| Malnes, 2016 | Average weekly distance | 37.1 (21) | |  |  |  | |  | |  | | |  | |  |
|  | **High Fitness** | 43.8 (16.4) | |  |  |  | |  | |  | | |  | |  |
|  | **Low Fitness** | 36.1 (25.6) | |  |  |  | |  | |  | | | 0.472 H vs. L | |  |
|  | Average weekly duration | 107 (62) | |  |  |  | |  | |  | | |  | |  |
|  | Time to exhaustion |  | | 11.4, *(10.5, 12.4)* | 12.5, *(11.4, 13.6)* |  | |  | |  | | | 0.069 | |  |
|  | **% gain - time to exhaustion** | | | | | | | | | | | | | | |
|  | **High Fitness** |  | |  | -2.5 *(-22.3, 17.3)* |  | |  | |  | | | 0.561 | |  |
|  | **Low Fitness** |  | |  | 14.3,*(4.1, 24.5)* |  | |  | |  | | | 0.028 | |  |
|  | Respiratory exchange ratio |  | | 1.27, *(1.21, 1.32)* | 1.25, *(1.20, 1.30)* |  | |  | |  | | | 0.272 | |  |
|  | Ventilation |  | | 119.2, *(106.2, 132.2)* | 119.8, *(104.5, 134.7)* |  | |  | |  | | | 0.755 | |  |
|  | *Mean (Range)* | **E-bike commute** | | | | **Passive commute** | | | | | | |  | |  |
| Page, 2017 | Average distance* | 10.31 (5.63, 20.92) | |  |  | 17.08 (5.63-29.12) | |  | |  | | |  | |  |
|  | Average ride time* | 6 (3-8) | |  |  | NA | |  | |  | | |  | |  |
|  | Average frequency | 1-2 | |  |  | NA | |  | |  | | |  | |  |
|  |  | **E-bike** | | | |  | |  | |  | | |  | |  |
| Peterman, 2016^+^ | Average ride time | 58.5 (15.2) | |  |  |  | |  | |  | | |  | |  |
|  | Average distance | 19.7 (8.8) | |  |  |  | |  | |  | | |  | |  |
|  | Average METh | 5.2 (2.1) | |  |  |  | |  | |  | | |  | |  |
|  | Absolute mean EE per ride | 420.1 (221.8) | |  |  |  | |  | |  | | |  | |  |
| *self-report measures of activity – included to provide indication of activity level reported  ^+^ results reported for days in which cycling was prescribed (i.e., 3 days a week for at least 40-minutes)  NA, not applicable, NC, not conducted, EE = energy expenditure (measured in Kcal), METh = metabolic equivalent hours, PA = physical activity measured in minutes per week; respiratory exchange ratio = ratio between amount of carbon dioxide produced and oxygen used  *Distance* (total and weekly) measured in kilometers; *Duration* (total and weekly) measured in minutes; *additional PA* measured in minutes; *time to exhaustion* measured in minutes; *MET-hours* measured as METscore/mins expressed in hours; *Absolute power* measured as Watts; *ventilation* measured in l/min; *Absolute mean EE per ride* measured as kcal. | | | | | | | | | | | | | | | |
